# A comprehensive multi-omics analysis uncovers the associations between gut microbiota and pancreatic cancer

**DOI:** 10.3389/fmicb.2025.1592549

**Published:** 2025-05-01

**Authors:** Yang Han, Biyang Cao, Jiayue Tang, Jing Wang

**Affiliations:** ^1^Medical Innovation Research Division, Chinese PLA General Hospital, Beijing, China; ^2^The First Medical Center, Chinese PLA General Hospital, Beijing, China; ^3^The Second Medical Center, Chinese PLA General Hospital, Beijing, China

**Keywords:** gut microbiota, metabolome, pancreatic cancer, single nucleotide polymorphisms, metagenomic binning

## Abstract

Pancreatic cancer is one of the most lethal malignant neoplasms. Pancreatic cancer is related to gut microbiota, but the associations between its treatment and microbial abundance as well as genetic variations remain unclear. In this study, we collected fecal samples from 58 pancreatic cancer patients including 43 pancreatic ductal adenocarcinoma (PDAC) and 15 non-PDAC, and 40 healthy controls, and shotgun metagenomic sequencing and untargeted metabolome analysis were conducted. PDAC patients were divided into five groups according to treatment and tumor location, including treatment-naive (UT), chemotherapy (CT), surgery combined with chemotherapy (SCT), Head, and body/tail (Tail) groups. Multivariate association analysis revealed that both CT and SCT were associated with increased abundance of *Lactobacillus gasseri* and *Streptococcus equinus*. The microbial single nucleotide polymorphisms (SNPs) densities of *Streptococcus salivarius*, *Streptococcus vestibularis* and *Streptococcus thermophilus* were positively associated with CT, while *Lachnospiraceae bacterium 2_1_58FAA* was positively associated with Head group. Compared with Tail group, the Head group showed positive associations with opportunistic pathogens, such as *Escherichia coli*, *Shigella sonnei* and *Shigella flexneri.* We assembled 424 medium-quality non-redundant metagenome-assembled genomes (nrMAGs) and 276 high-quality nrMAGs. In CT group, indole-3-acetic acid, capsaicin, sinigrin, chenodeoxycholic acid, and glycerol-3-phosphate were increased, and the accuracy of the model based on fecal metabolites reached 0.77 in distinguishing healthy controls and patients. This study identifies the associations between pancreatic cancer treatment and gut microbiota as well as its metabolites, reveals bacterial SNPs are related to tumor location, and extends our knowledge of gut microbiota and pancreatic cancer.

## Graphical abstract

The analysis process of this study.

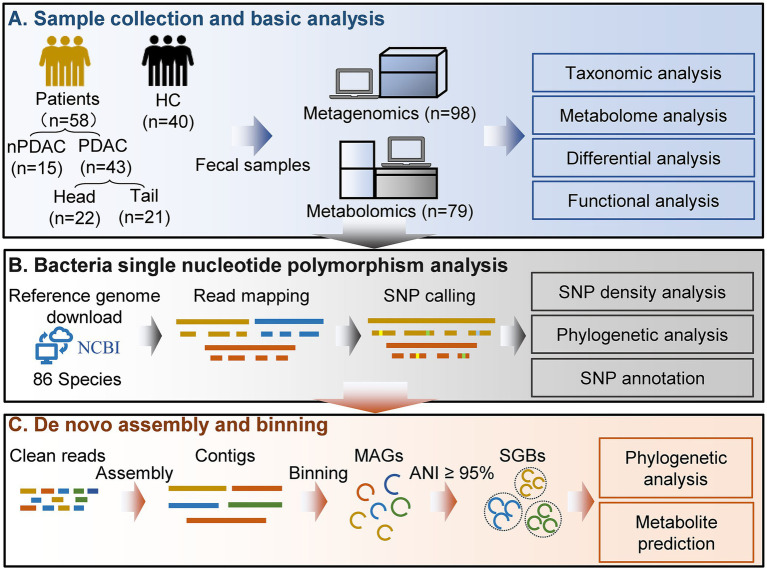

## Introduction

Pancreatic cancer is one of the most lethal malignant neoplasms, and the 5-year survival rate is less than 5% (Nagata et al., 2022). Over 80% of pancreatic malignancies are pancreatic ductal adenocarcinoma (PDAC) (Zhou et al., 2021). Currently, surgical resection remains the only potentially curative treatment for pancreatic cancer, and the addition of adjuvant chemotherapy has been shown to improve survival rates (McGuigan et al., 2018). Studies have revealed that pancreatic cancer is associated with age, sex, ethnicity, blood group and gut microbiota (McGuigan et al., 2018). Previous study indicated that PDAC group had a lower gut microbial Shannon Index compared with healthy controls (Hong et al., 2024), and gut microbiota as a biomarker for pancreatic cancer detection has been reported (Sidiropoulos et al., 2024). Moreover, microbial composition and function can influence the onset and progression of pancreatic cancer (Cheng et al., 2025). In addition, higher fecal *Lactobacillus* abundance correlated with improved progression-free and overall survival of PDAC (Liang et al., 2025). While evidence supporting a potential link between gut microbiota and the progression of pancreatic cancer, the relationship between gut microbiota and treatment outcomes remains underexplored. The role of gut microbiota in the treatment of pancreatic cancer has yet to be fully established. Regarding tumor location, PDAC is typically classified into pancreatic head and body/tail cancer, with several studies highlighting differences in prognosis between these subtypes (Yun et al., 2024). Although gut microbiota of patients with pancreatic head cancer has been characterized using 16S rRNA gene pyrosequencing (Mei et al., 2018), the limitations of this method have precluded a more detailed analysis at the species or strain level.

In addition, the majority of current association studies are primarily based on microbial abundance, which is a fundamental characteristic of microbial communities. However, abundance-based studies alone cannot comprehensively elucidate the complex relationship between gut microbiota and pancreatic cancer. Single nucleotide polymorphisms (SNPs) are prevalent in microbiome, and SNPs within gut microbial genome can influence bacterial functions, such as drug metabolism and response (Olm et al., 2021; Shi et al., 2022). Some studies have established associations between microbial SNPs and various conditions such as host body mass index (Zahavi et al., 2023), colorectal cancer (Ma et al., 2021a) and liver cirrhosis (Chen et al., 2021). However, the potential associations between microbial SNPs and pancreatic cancer have not been revealed.

Since the majority of microorganisms remain uncultured and lack complete reference genomes, taxonomic annotation of the gut microbiome often fails to capture many unknown taxa due to database incompleteness (Saheb Kashaf et al., 2021). Additionally, traditional microbial genes profiling methods are difficult to assign specific gene to precise microbial species, posing a challenge for species-level functional analysis. Currently, *de novo* assembly and metagenomic binning approaches effectively reconstruct metagenome-assembled genomes (MAGs) from metagenomic sequencing short reads for organisms that have not yet been isolated and cultured. However, the reference genome of gut microbiota for pancreatic cancer has not been established. In addition, fecal metabolites produced by microbes are typically key mediators of host–microbe interactions. Metabolites derived from microbes hold considerable promise for the risk assessment and prognostication of pancreatic cancer (León-Letelier et al., 2024). However, associations between different treatments for pancreatic cancer and gut microbiota has not been revealed. Integrated analysis of microbiome and fecal metabolome is essential to identify microbial metabolites associated with pancreatic cancer treatments.

Here, we collected 98 fecal samples from 40 healthy controls (HC) and 58 pancreatic cancer patients, including 43 patients with pancreatic ductal adenocarcinoma (PDAC) and 15 with non-pancreatic ductal adenocarcinoma (nPDAC). PDAC patients were divided into three groups based on treatments: treatment-naive (UT, *n* = 15), chemotherapy (CT, *n* = 19), surgery combined with chemotherapy (SCT, *n* = 9). All CT or SCT patients who received chemotherapy were treated with 5-FU-based protocols. The minimum interval between the initiation of chemotherapy and sample collection was 20 days, and the shortest interval between surgery and sampling was 83 days, ensuring sufficient time to observe changes of gut microbiota. Additionally, PDAC patients were classified by tumor location into pancreatic head cancer (Head, *n* = 22) and pancreatic body/tail cancer (Tail, *n* = 21) (Table 1). Metagenomic sequencing and untargeted metabolome analysis were performed on fecal samples. Furthermore, microbial SNPs analysis was conducted to establish associations between SNPs and pancreatic cancer. MAGs were constructed separately for HC and pancreatic cancer patients by metagenomic binning to recover genomes for organisms that have yet to be isolated and cultured.

**Table 1:** Demographic and clinical details of samples.

Variable		Pancreatic cancer group				HC (*n* = 40)	PDAC (*n* = 43)	nPDAC (*n* = 15)	*p*-value	Effect size (Cohen’s d)
Age	44.95 ± 17	58.12 ± 8.05	55.8 ± 8.39	<0.001	−1.004 (−1.428~−0.574)
Gender (Female)	55.00% (22)	34.88% (15)	53.33% (8)	0.598	
BMI	25.07 ± 3.72	22.99 ± 3.34	23.79 ± 3.45	0.011	0.534 (0.123~0.943)
Hypertension	0	37.21% (16)	26.67% (4)	<0.001	
Diabetes	0	25.58% (11)	20.00% (3)	<0.001	
Coronary heart disease	0	9.30% (4)	20.00% (3)	0.023	
Treatments					
UT	-	34.88% (15)	86.66% (13)		
CT	-	44.19% (19)	6.67% (1)		
SCT	-	20.93% (9)	6.67% (1)		
Tumor location					
Pancreatic head	-	51.16% (22)	-		
Pancreatic body/tail	-	48.83% (21)	-		

HC, healthy control; PDAC, pancreatic ductal adenocarcinoma; nPDAC, non-pancreatic ductal adenocarcinoma; BMI, body mass index; UT, treatment-naïve; CT, chemotherapy; SCT, surgery combined with chemotherapy. *p* value and effect size were calculated by comparing HC group and pancreatic cancer group.

## Methods

### Sample collection

The stool samples were collected at Chinese PLA General Hospital from participants who voluntarily enrolled in the study and provided informed consent. Our research protocol was approved by the Ethics Committee of the Chinese PLA General Hospital (S2024-015-01). Fresh stool specimens were collected in the morning following an overnight fast and were immediately flash-frozen in liquid nitrogen prior to storage at −80°C. Stool genomic DNA was extracted using a magnetic stool kit (TIANGEN, China), which effectively disrupts various components and ensures the integrity of DNA through the use of glass beads. To avoid potential batch effects, fecal genomic DNA extraction kits of the same brand and same production batch were used for all samples. In addition, fecal genomic DNA was extracted and sequenced in the same batch for all samples. Covariates including age, sex, body mass index, hypertension, diabetes and coronary heart disease were accounted for to minimize potential confounding factors. Diet is an important factor influencing the gut microbiota. In this study, the controls and patients were both included from the inpatients of our hospital. The control group were all inpatients due to fractures. During the hospitalization, the hospital provided a basically consistent diet to all inpatients every day, thereby effectively controlling the potential differences in diet. Inclusion criteria for this study: (1) Participants had not received antibiotics or probiotics within 3 months before sampling; (2) Participants had no other gastrointestinal cancers; (3) Participants were Han Chinese living in Beijing, China; (4) Patients in the treatment group received treatment for at least 20 days; (5) Participants had no family history of pancreatic cancer. (6) The healthy controls did not suffer from any other diseases or tumors.

### Metagenomic sequencing

#### Quality control and assembly

Shotgun paired-end sequencing was performed on all DNA samples using the HiSeq 2,500 platform at Novogene Co., Ltd. (China). Quality control of the raw sequencing data was conducted to obtain high-quality clean reads using Readfq (v8.0). First, reads containing more than 40 consecutive low-quality bases (quality score < 38) were discarded. Second, reads with more than 10 “*N*” bases and reads with adapter contamination exceeding 15 bp were removed. To eliminate host sequences, the remaining reads were aligned to the human genome (GRCh37) using Bowtie2 (v2.2.4) with parameters “--end-to-end, --sensitive, -I 200, -X 400.” Finally, the clean reads were assembled into scaffolds using MEGAHIT (v1.0.4) with parameters “--presets meta-large,” and scaffolds without “*N* “are obtained by breaking from the “*N* “junction.

#### Gene predicting and abundance analysis

Open reading fragments (ORFs) on scaffolds were predicted using MetaGeneMark (v2.10) with default parameters, and ORFs shorter than 100 nucleotides were excluded. To construct a non-redundant initial gene catalog, redundant sequences in the predicted ORFs were removed using CD-HIT (v4.5.8) with parameters “-c 0.95, -G 0, -aS 0.9, -g 1, -d 0.” Clean reads after quality control and assembly were aligned to initial gene catalog to calculate the number of aligned reads for each gene using Bowtie2 (v2.2.4) with parameters “--end-to-end, --sensitive, -I 200, -X 400,” and genes with fewer than two aligned reads were excluded. The resulting gene catalog was then aligned to the NCBI NR database (v2018.01) using DIAMOND (v0.9.9.110) with parameters “blastp, -e 1e-5,” and the Lowest Common Ancestor algorithm was applied to determine the taxonomic information. The abundance of a specific taxonomical level in a sample was calculated as the sum of the abundances of genes assigned to that level. To determine functional hierarchies and their corresponding relative abundances, the gene catalog was aligned to the Kyoto Encyclopedia of Genes and Genomes (KEGG) database (v2018.01) using DIAMOND.

### Single nucleotide polymorphisms analysis

In total, the reference genomes of 86 species were downloaded from the NCBI database. Following quality control, the reads were aligned to the respective reference genomes using the Burrows-Wheeler Aligner (BWA, v0.7.8). SNP calling was performed using BCFtools (v1.15.1) with parameters “-m 3 -F 0.0002 -C 50.” To reduce false-positive calls, the mpileup2snp command of VarScan (v2.4.4) was utilized with parameters “--min-coverage 10 --min-reads2 4 --min-var-freq 0.2 --*p*-value 0.05.” Only SNPs identified by both BCFtools and VarScan2 were retained for subsequent analysis. Given the positive correlation between SNPs frequency and sequencing depth, the normalized number of SNPs based on sequencing depth was used to calculate SNP density (Ma et al., 2021b). The number of normalized SNPs was equal to the original number of SNPs divided by the sequencing depth of the sample. The SNPs annotation was conducted using SnpEff (v4.3) (Cingolani et al., 2012), and the annotation results included synonymous mutations, missense mutations and other types.

### Phylogenetic analysis of strains based on SNPs

We calculated the p-distance matrix using VCF2Dis (v1.52)^1^ based on Variant Call Format input. The distance matrix was subsequently utilized to construct a neighbor-joining phylogenetic tree via Fastme (v2.0) (Lefort et al., 2015) online software. The resulting tree was visualized using the R package ggtree (v3.12.0) (Yu, 2022).

### Microbial genome reconstruction

#### Metagenome assembly and binning

The microbial genome reconstruction was conducted using MetaWRAP (v1.3.2) (Uritskiy et al., 2018) following the recommended analysis pipeline. First, clean sequencing data from both disease and healthy control groups were separately assembled using the metawrap-assembly module with MEGAHIT. Second, the assemblies were binned using the metawrap-binning module employing metaBAT2 (Kang et al., 2015), MaxBin2 (Wu et al., 2016) and CONCOCT (Alneberg et al., 2014), respectively, and the minimum contig length used for bin construction was set as the default value. Third, the MAGs were refined using the bin_refinement module, and the completeness and contamination levels were evaluated with CheckM (Parks et al., 2015). Only MAGs exhibiting greater than 50% completeness and less than 10% contamination were retained for further analysis.

#### Abundance estimation and taxonomy annotation

The abundance of MAGs across the samples was estimated using the quant_bins module of MetaWRAP. The abundance was calculated with Salmon (Patro et al., 2017) as “genome copies per million reads.” Taxonomic classification of each MAG was determined using the Classify_bins module, which employes Taxator-tk to assign taxonomy to individual contigs and subsequently integrates these assignments to estimate the overall taxonomic composition of the MAG. For taxonomic annotation, both the NCBI_nt BLAST database (v2023.11) and the NCBI taxonomy database (v2023.11) were utilized.

#### Dereplication and phylogenetic analysis of MAGs

The species-level genome bins (SGBs) were clustered at a 95% average nucleotide identity (ANI) threshold using the “dereplicate” function in dRep (v3.4.5) (Olm et al., 2017). Dereplication of all MAGs was conducted to obtain non-redundant MAGs (nrMAGs) at a 99% ANI threshold through a two-step process. First, MAGs were clustered into primary clusters based on a 90% ANI threshold. Second, these primary clusters were further clustered into secondary clusters using a 99% ANI threshold with a minimum overlap of 30%. Phylogenetic analysis of the nrMAGs was performed using PhyloPhlAn (v3.0.67) (Asnicar et al., 2020), and the resulting tree was visualized using the web-based application iTOL (Letunic and Bork, 2024).

#### Prediction of the secondary metabolites of MAGs

The secondary metabolites associated with each MAG were predicted using antiSMASH (v7.1.0) (Blin et al., 2023) with default settings. The antiSMASH is a widely recognized tool designed to identify gene clusters involved in the biosynthesis of microbial secondary metabolites.

### UHPLC-MS/MS analysis

Untargeted metabolomic analysis of stool samples was conducted at Novogene Co., Ltd. (China). An 80% methanol solution was added to the stool samples, which were then vortexed and incubated in a water bath at 4°C for 5 min. Subsequently, the samples were centrifuged at 15,000 × g for 20 min at 4°C. A specific volume of the supernatant was diluted with a 53% methanol solution and subjected to another centrifugation at 15,000 × g for 20 min at 4°C. The resulting supernatant was analyzed by LC–MS/MS. Chromatographic separation was achieved on a Hypesil Gold column using a linear gradient over 17 min at a flow rate of 0.2 mL/min. For positive ion mode, the mobile phases consisted of eluent A (0.1% formic acid in water) and eluent B (methanol). In negative ion mode, eluent A (5 mM ammonium acetate in water) and eluent B (methanol) were used as the mobile phases.

### Machine learning

The eXtreme Gradient Boosting (XGBoost) algorithm was utilized for classifying healthy controls and pancreatic cancer patients based on metagenomic and metabolomic data using the R package xgboost (v1.6.0) (Chen and Guestrin, 2016). For the reproducibility of the models, we employed multiple random seeds and 10-fold cross-validation strategy. The receiver operating characteristic (ROC) curve visualization and the area under the ROC curve (AUC) value calculation were conducted using the R package ROCR (v1.0.11). For the training and validation datasets, a random selection process allocated 70% of the samples to the training set and 30% to the validation set. To determine the optimal number of iterations, 25 repetitions of 10-fold cross-validation were performed.

### Statistical analysis

The principal coordinate analysis (PCoA) was conducted using the R package ape (v5.7-1). To identify multivariable associations between phenotypes and microbial features, including microbial species, functional pathways, and SNP density, we employed R package MaAsLin2 (v1.6.0) (Mallick et al., 2021), which relies on general linear models to accommodate most microbiome study designs, including support for multiple covariates. To account for potential confounding factors, random effects were included as covariates, specifically gender, age, body mass index, hypertension, diabetes, and coronary heart disease. The *p* values were adjusted using the Benjamini-Hochberg method to control the false discovery rate (FDR), with a default significance threshold set at 0.25. The demographical and clinical indicators of HC group and pancreatic cancer group were compared using JASP (0.19.3) software. Continuous variables satisfying a normal distribution were tested using the Student’s t-test, otherwise Mann–Whitney U test was used. Effect size and 95% confidence intervals were calculated with JASP. The Chi-square test was used for categorical variables.

Orthogonal partial least squares discriminant analysis (OPLS-DA) was conducted on the metabolomic data obtained from both positive and negative ion modes after log transformation and UV-scaling using SIMCA 14.1 software. A permutation test (*n* = 200) was performed to assess the model’s robustness and validity. Variable importance in projection (VIP) values were calculated using SIMCA 14.1 software to identify significant variables. The Wilcoxon rank sum test was applied to evaluate differences in metabolite levels, with *p* values adjusted for multiple testing using the Benjamini-Hochberg method (q value). Metabolites with q < 0.05 and VIP > 1 were identified as differential metabolites for further analysis. Visualization of differential metabolites was achieved using the R package pheatmap (v1.0.12). Pearson correlation analysis was conducted between the top 10% most abundant microbes and metabolites using the R package psych (v2.4.3), with *p* values corrected for multiple comparisons using the Benjamini-Hochberg method (q value). Correlation coefficients with |r| > 0.6 and q < 0.05 were visualized using Cytoscape (v3.8.2). Fisher’s exact test was performed using SPSS (v25) to examine the associations between phylogenetic clusters and study groups.

## Results

### Gut microbial composition in pancreatic cancer patients

**Figure 1:**
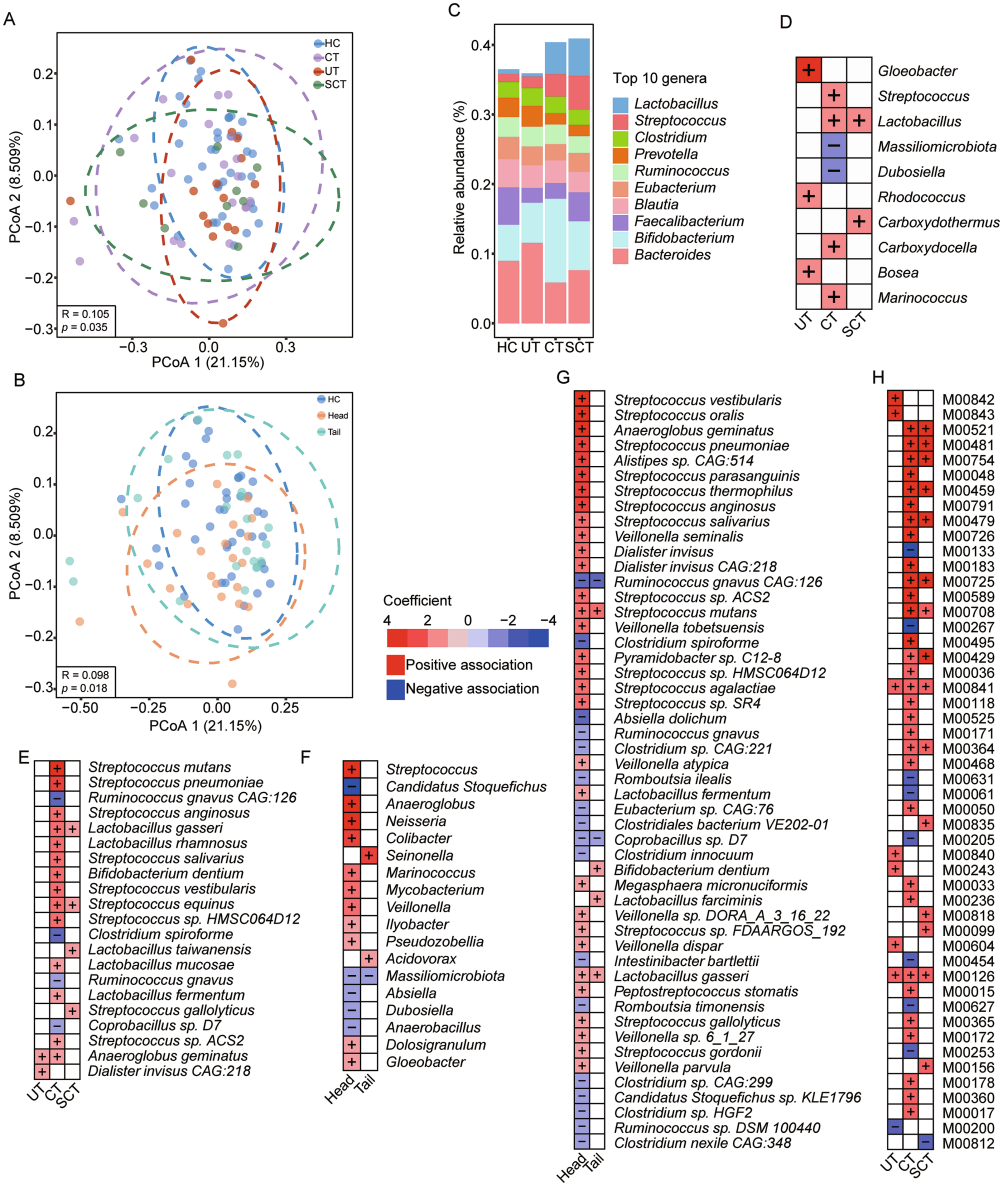
Differences of gut microbial composition across groups. **(A)** PCoA result of gut microbial composition among HC, UT, CT and SCT groups. **(B)** PCoA result of gut microbial composition among HC, Head and Tail groups. **(C)** The relative abundance of the top 10 genera in each group. **(D)** Analysis of associations between microbial genera and groups with the HC as the reference group using MaAslin2. **(E)** Analysis of associations between microbial species and groups with the HC as the reference group using MaAslin2. **(F)** Analysis of associations between microbial genera and groups with the HC as the reference group using MaAslin2. **(G)** Analysis of associations between microbial species and groups with the HC as the reference group using MaAslin2, and the top 50 significant associations are displayed. **(H)** Analysis of associations between microbial KEGG modules and groups with the HC as the reference group using MaAslin2, and the top 50 significant associations are displayed.

To investigate the differences in microbial composition across groups, principal coordinate analysis (PCoA) was conducted. Results indicated no significant differences among HC, PDAC and nPDAC groups over the first two dimensions (ANOSIM test, *p* = 0.581). In PDAC and HC groups, distinct clusters were observed among UT, CT, SCT and HC groups (Anosim test, R = 0.105, *p* = 0.035, Figure 1A), while no significant differences were found among UT, CT and SCT groups (Anosim test, *p* = 0.182). Notably, in PDAC and HC groups, significant clustering differences were observed among HC, Head and Tail groups (Anosim test, R = 0.098, *p* = 0.018, Figure 1B), with a significant difference also noted between the Head and Tail groups (Anosim test, R = 0.043, *p* = 0.039, Figure 1B). Analysis of the top 10 abundant genera revealed that *Lactobacillus*, commonly used as a probiotic due to its promising applications in intestinal health and disease (Huang et al., 2022), was positively associated with CT and SCT groups but not with UT (Figures 1C,D). Previous studies have highlighted the potential of *Lactobacillus* spp. in ameliorating pancreatic cancer and modulating gut microbial homeostasis (Zhu et al., 2023). *Streptococcus* showed a specific positive association with CT group, while *Gloeobacter*, *Rhodococcus* and *Bosea* were positively associated with UT group. In contrast, *Dubosiella*, which is positively correlated with short-chain fatty acids (Fang et al., 2023), exhibited a specific negative association with CT group.

Setting HC as the reference group, we further investigated the associations between disease groups and specific microbial species. The CT group exhibited increased abundances of *Streptococcus* spp. and *Lactobacillus* spp., such as *Streptococcus pneumoniae*, a cause agent of community-acquired pneumonia (Weiser et al., 2018), and *Streptococcus mutans*, which is predominantly observed in patients with underlying cardiovascular conditions (Nomura et al., 2020; Figure 1E). Conversely, the CT group showed decreased abundances of *Ruminococcus gnavus*, *Ruminococcus gnavus CAG:126* and *Clostridium spiroforme*. Both CT and SCT groups were associated with increased abundances of *Lactobacillus gasseri* and *Streptococcus equinus*, which has been implicated in colorectal cancer and infective endocarditis (Kaiki et al., 2021).

To investigate the associations between microbes and the Head or Tail groups, HC was designated as the reference group. Results revealed positive associations between *Streptococcus* and *Anaeroglobus* with Head group, while *Massiliomicrobiota* and *Candidatus Stoquefichus* showed negative associations (Figure 1F). *Seinonella* and *Acidovorax* exhibited positive associations with the Tail group. At the species level, out of 810 abundant species, 51 species showed significant associations with either Head or Tail group, and the top 50 significant associations were displayed (Figure 1G). Specifically, *Streptococcus vestibularis* and *Streptococcus oralis* were positively associated with Head group, whereas *Clostridium spiroforme* and *Absiella dolichum* showed negative associations. Only six significant associations were identified between species and Tail group. Setting Head group as the reference, nine negative associations were found between species and Tail group, including opportunistic pathogens such as *Escherichia coli*, *Shigella sonnei*, *Shigella flexneri* and *Shigella dysenteriae*. Previous study has reported the association of *Escherichia coli* with the development of PDAC (Wei et al., 2019). Also, *Veillonella* spp., such as *Veillonella parvula*, *Veillonella* sp. *oral taxon 158* and *Veillonella tobetsuensis*, were significantly negatively associated with Tail group.

### Alterations of microbial function in pancreatic cancer patients

The KEGG module analysis revealed that the UT, CT and SCT groups were all characterized by an increased abundance of “Tetrahydrofolate biosynthesis” (M00841, M00126) (Figure 1H). In contrast, the CT group exhibited a decreased abundance of “Polyamine biosynthesis” (M00133), “D-Glucuronate degradation” (M00061) and “beta-Lactam resistance” (M00627). The SCT group showed increased abundance of “Pyocyanine biosynthesis” (M00835), “Sphingosine biosynthesis” (M00099) and “Cytochrome c oxidase” (M00156).

**Figure 2:**
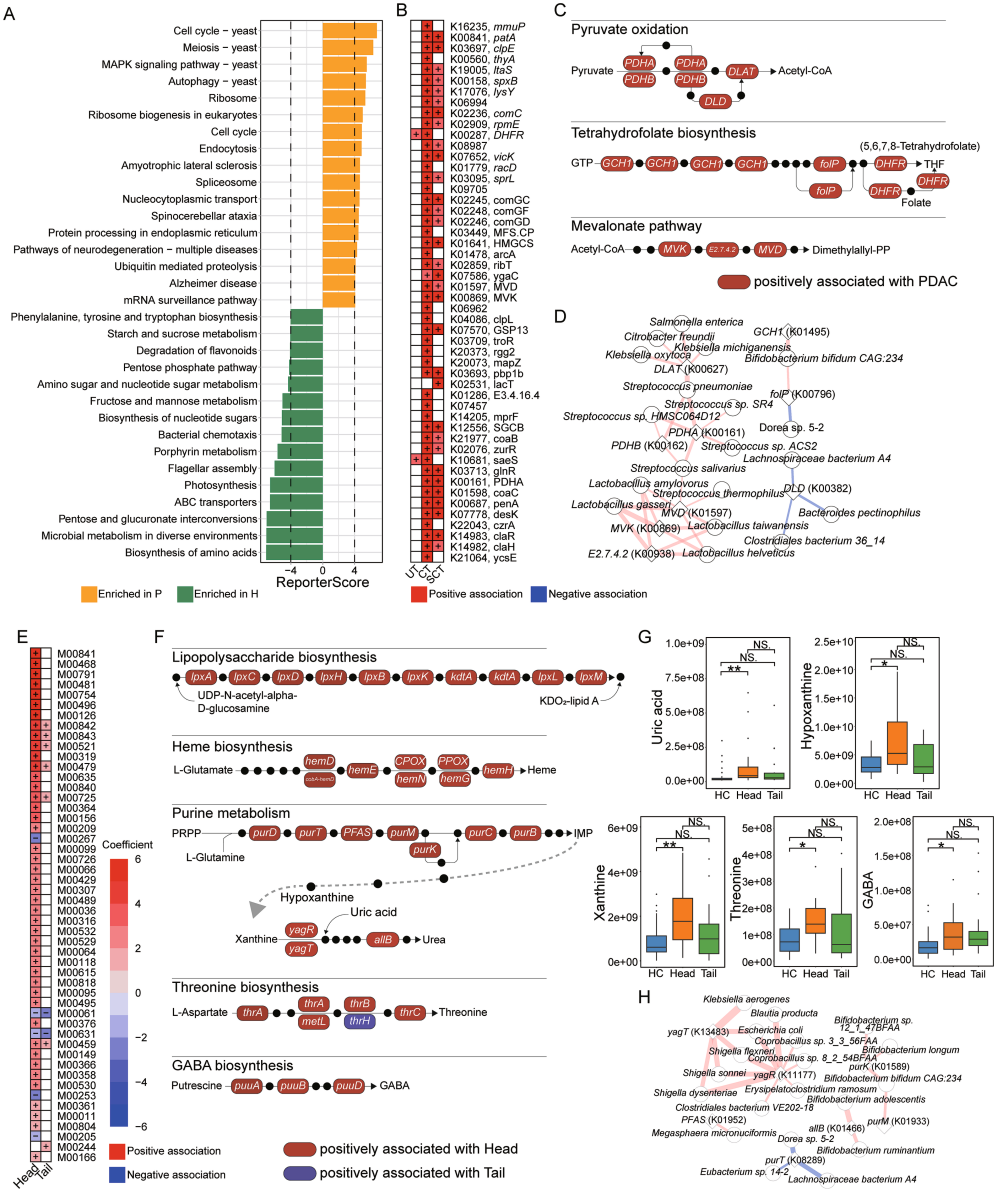
Microbial function and correlation analysis. **(A)** KO enrichment analysis for HC and patient groups. **(B)** Analysis of associations between microbial KO genes and groups with the HC as the reference group using MaAslin2, and the top 50 significant associations are displayed. **(C)** Microbial KEGG module analysis for “Pyruvate oxidation,” “Tetrahydrofolate biosynthesis,” “Tetrahydrofolate biosynthesis.” Red box represents microbial KO genes positively associated with PDAC group. Black dot represents an intermediate metabolite. **(D)** Pearson correlation analysis between microbial KO genes and species (|r| > 0.6). Red line represents positive correlation, and blue line represents negative correlation. The thickness of the line represents the strength of the correlation. **(E)** Analysis of associations between microbial KEGG modules and groups with the HC as the reference group using MaAslin2, and the top 50 significant associations are displayed. **(F)** Microbial KEGG module analysis for “Lipopolysaccharide biosynthesis,” “Heme biosynthesis,” “Purine metabolism,” “Threonine biosynthesis,” and “GABA biosynthesis.” Red box represents microbial KO genes positively associated with the Head group, and blue box represents microbial KO genes positively associated with the Tail group. Black dot represents an intermediate metabolite. **(G)** UHPLC–MS/MS analysis for fecal uric acid, hypoxanthine, xanthine, threonine and GABA levels. **(H)** Pearson correlation analysis between microbial KO genes involved in purine metabolism and species (|r| > 0.6). Red line represents positive correlation, and blue line represents negative correlation. The thickness of the line represents the strength of the correlation.

The enrichment analysis of KO genes indicated that pathways, such as “Cell cycle,” “MAPK signaling pathway,” “Autophagy” and “Endocytosis,” were significantly enriched in PDAC patients. Conversely, pathways like “Biosynthesis of amino acids,” “Porphyrin metabolism,” “Pentose phosphate pathway” and “Phenylalanine, tyrosine and tryptophan biosynthesis” were predominantly enriched in HC (Figure 2A). A total of 265 KO genes were associated with UT, CT or SCT groups, with 217 and 103 KO genes specifically linked to CT and SCT groups, respectively, and 71 KO genes shared between CT and SCT. The top 50 features with significant associations are illustrated (Figure 2B). Further analysis revealed significant associations between PDAC group and increased abundance of KO genes involved in microbial metabolic pathways, particularly “Mevalonate pathway,” “Pyruvate oxidation” and “Tetrahydrofolate biosynthesis” (Figure 2C). Notably, the CT and SCT groups showed elevated levels of *PDHA* (K00161) and *PDHB* (K00162), which are key enzymes in pyruvate oxidation, as well as *MVK* (K00869), *E2.7.4.2* (K00938) and *MVD* (K01597), which are involved in the mevalonate pathway. However, these changes were not observed in UT group, suggesting their potential modulations by treatments. Pearson correlation analysis demonstrated that KO genes involved in “Mevalonate pathway” and “Pyruvate oxidation” were significantly positively correlated with *Lactobacillus* spp. and *Streptococcus* spp., respectively, indicating that alterations in the abundance of these species may play a critical role in microbial pyruvate oxidation and mevalonate pathway in CT and SCT groups (|r| > 0.6, Figure 2D).

The associations between KEGG modules and the Head or Tail groups were further analyzed. Multi-group analysis revealed a significant positive correlation between the Head group and the KEGG modules “Tetrahydrofolate biosynthesis” (M00841, M00126, M00840), “Tetracycline resistance” (M00635) and “Sphingosine biosynthesis” (M00099) (Figure 2E). We further examined the relationships between KO genes and the Head or Tail groups. Notably, positive associations were observed between the Head group and KO genes involved in “Lipopolysaccharide biosynthesis,” “Heme biosynthesis,” “Purine metabolism,” “Threonine biosynthesis” and “GABA biosynthesis” (Figure 2F). We further quantified the levels of intermediate metabolites from “Purine metabolism,” “Threonine biosynthesis” and “GABA biosynthesis” in fecal samples. Consistently, uric acid, hypoxanthine and xanthine levels were significantly elevated in the Head group, while no differences were observed between the HC and Tail groups (Figure 2G). Additionally, threonine and GABA levels in feces were significantly increased in the Head group compared to HC. These results suggested that the increased abundance of microbial KO genes associated with “Purine metabolism,” “Threonine biosynthesis” and “GABA biosynthesis” may be related to the elevation of fecal metabolites produced by these pathways. Pearson correlation analysis was conducted to identify species associated with altered KO genes involved in “Purine metabolism” (|r| > 0.6, Figure 2H). The results indicated that *yagT* (K13483) and *yagR* (K11177) were positively correlated with *Shigella* spp. and *Escherichia coli*, while *purK* (K01589), *allB* (K01466) and *purM* (K01933) were positively correlated with *Bifidobacterium* spp. Speculatively, the increased abundance of *Shigella* spp. and *Escherichia coli* in the Head group may be related to the elevated KO genes involved in purine metabolism and higher uric acid levels in feces.

### Single nucleotide polymorphism of microbes in pancreatic cancer patients

**Figure 3:**
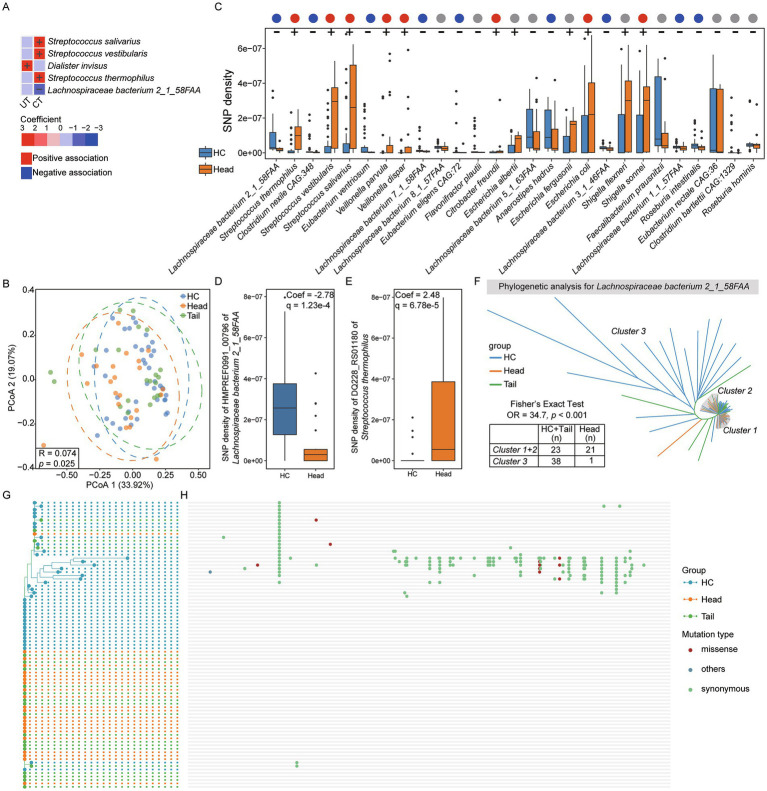
Single nucleotide polymorphisms analysis. **(A)** Setting HC as the reference group, association analysis between each group and microbial SNPs densities. **(B)** PCoA result for HC, Head and Tail groups. **(C)** Setting HC as the reference group, significant associations between the Head group and microbial SNPs densities (q < 0.25). The positive associations were indicated by “+,” and the negative associations were indicated by “-.” The red, blue and gray dots represent positive, negative, and no associations between Head group and microbes in abundance, respectively. **(D)** The most significant gene of *Lachnospiraceae bacterium 2_1_58FAA* associated with Head group. **(E)** The most significant gene of *Streptococcus thermophilus 2_1_58FAA* associated with Head group. **(F)** Phylogenetic analysis for *Lachnospiraceae bacterium 2_1_58FAA* genome based on all genes across 83 samples. G and H. Phylogenetic analysis for isoleucyl-tRNA synthetase (HMPREF0991_00796) of *Lachnospiraceae bacterium 2_1_58FAA* **(G)** and single nucleotide mutation sites analysis **(H)**. The red dots represent missense mutations, green dots represent synonymous mutations and blue dots represent other mutation types.

Furthermore, single nucleotide polymorphisms (SNPs) analysis was conducted across 86 species, and the normalized SNP densities were calculated for each species. PCoA results indicated no significant differences in SNP density among HC, UT, CT and SCT (*p* > 0.05). Association analysis revealed that the SNP densities of *Streptococcus salivarius*, *Streptococcus vestibularis* and *Streptococcus thermophilus* were positively associated with CT, while *Lachnospiraceae bacterium 2_1_58FAA* exhibited an inverse relationship (Figure 3A). The SNP density of *Dialister invisus* was positively associated with UT. Additionally, PCoA result showed a weak separation among HC, Head and Tail groups (Anosim test, R = 0.074, *p* = 0.025, Figure 3B). Further association analysis identified 27 out of 86 species significantly associated with the Head group, including 10 species not previously identified through abundance analysis (Figure 3C). However, no associations were found between microbial SNP density and the Tail group. *Lachnospiraceae bacterium 2_1_58FAA* showed the highest significance in SNP density between HC and Head groups. Moreover, *Faecalibacterium prausnitzii*, a highly abundant gut microbe, showed no differences in abundance but exhibited lower SNP density in Head group compared to HC.

To further identify the significant gene of *Lachnospiraceae bacterium 2_1_58FAA* associated with the Head group in terms of SNP density, association analysis was conducted between microbial gene SNP density and the Head group. Among the 3,578 genes analyzed, 3,570 genes exhibited a significant association with the Head group (q < 0.25). Notably, isoleucyl-tRNA synthetase (locus_tag: HMPREF0991_00796; GenBank: EGN49576.1) ranked highest in significance (Figure 3D). Additionally, out of 1,109 genes from *Streptococcus thermophilus*, 1,006 showed significant associations with the Head group, with DQ228_RS01180 gene showed the highest significance (Figure 3E).

To investigate the phylogenetic characteristics of *Lachnospiraceae bacterium 2_1_58FAA* across 83 samples, we constructed a phylogenetic tree based on SNPs (Figure 3F). The resulting tree was divided into three distinct clusters: *Cluster 1* and *Cluster 2* predominantly comprised members from the Head group, while *Cluster 3* mainly consisted of individuals from the HC and Tail groups. Fisher’s exact test confirmed a significant association between the phylogenetic characteristics and the Head group (OR = 34.7, *p* < 0.001). Furthermore, we analyzed the SNPs in the isoleucyl-tRNA synthetase gene of *Lachnospiraceae bacterium 2_1_58FAA*. A phylogenetic tree was constructed based on SNPs, and their distributions were visualized (Figures 3G,H). Most SNPs were observed in HC group, whereas almost all individuals in Head group have no SNPs. Importantly, the majority of SNPs are synonymous mutations, indicating that they do not alter the encoded amino acids. SNPs analysis results suggest a strong association between microbial SNPs and the Head group.

### A high-quality microbial genome catalog for pancreatic cancer

**Figure 4:**
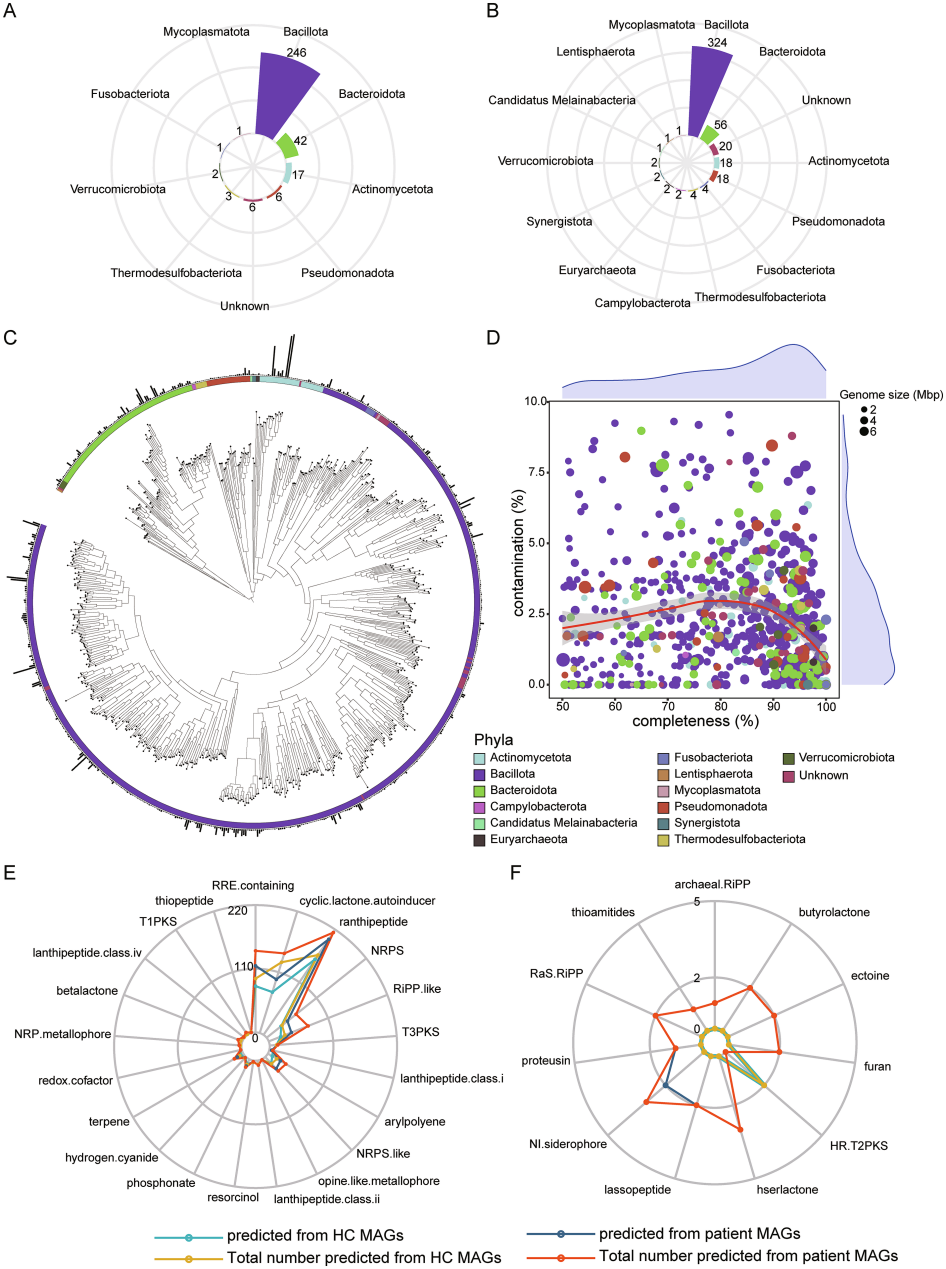
*De novo* assembly and binning. **(A)** A total of 324 MAGs annotated to each phylum from HC dataset assembly. **(B)** A total of 455 MAGs annotated to each phylum from patient dataset assembly. **(C)** Phylogenetic analysis for 723 nrMAGs. **(D)** The distribution of completeness and contamination across nrMAGs. The color of point represents phylum, and the size of point represents the genome size of nrMAGs. **(E)** Microbial secondary metabolites predicted using antiSMASH based on all MAGs. **(F)** Specific microbial secondary metabolites predicted based on HC or patient MAGs.

To construct a high-quality microbial genome catalog, we performed metagenomic assembly and binning separately on HC and patient groups. A total of 324 MAGs were recovered from HC group, with an average completeness of 82.65%, contamination of 2.32%, genome size of 2.24 megabases (Mb), and N50 of 18.75 kilobases (kb). MAGs were obtained using thresholds of ≥ 50% genome completeness and ≤ 10% contamination. All 324 MAGs were retained at an average nucleotide identity (ANI) threshold of 95% to generate species-level genome bins (SGBs). Among these, six SGBs were classified as unknown SGBs that could only be annotated to the Bacteria kingdom or lower taxonomic levels (Figure 4A). In patient group, a total of 455 MAGs were recovered, with an average completeness of 80.21%, contamination of 2.34%, genome size of 2.14 megabases (Mb) and N50 of 19.44 kb. All 455 MAGs were SGBs, among which 20 SGBs were classified as unknown SGBs (Figure 4B). Moreover, 86% of the SGBs in the patient group belonged to the phyla Bacillota and Bacteroidota.

We further integrated 779 SGBs and dereplicated them using an ANI threshold of 99% to obtain a final set of non-redundant MAGs (nrMAGs) with strain-level resolution. Ultimately, a total of 723 nrMAGs were retained. Among these, 424 nrMAGs were classified as medium-quality (50% ≤ completeness < 90% and contamination ≤ 5%), while 276 nrMAGs satisfied the high-quality criteria (completeness ≥ 90% and contamination ≤ 5%). Based on 723 nrMAGs, phylogenetic tree was constructed and displayed (Figure 4C), and the genome size, contamination level, and completeness of nrMAGs were displayed (Figure 4D).

To investigate the metabolic functions of MAGs, the secondary metabolites produced by 779 MAGs were predicted. Results revealed that 22 metabolites could be produced by MAGs assembled from HC group, whereas 31 metabolites were identified in MAGs from the patient group. Among these, 21 metabolites were common to both groups (Figure 4E). The top three prevalent metabolites across the majority of MAGs were ranthipeptide, RRE-containing and cyclic-lactone-autoinducer. HR-T2PKS was exclusively predicted in MAGs from HC group, while 10 metabolites, such as thioamitides, archaeal-RiPP and hserlactone, were uniquely predicted in MAGs from the patient group (Figure 4F). Notably, thioamitides and lassopeptide, which have significant potential for anticancer drug development (Cheng and Hua, 2020; Hu et al., 2022), as well as ectoine, a novel anti-inflammatory and tissue-protective compound (Bethlehem and van Echten-Deckert, 2021), were specifically predicted in MAGs from the patient group.

### Fecal metabolome analysis across groups

**Figure 5:**
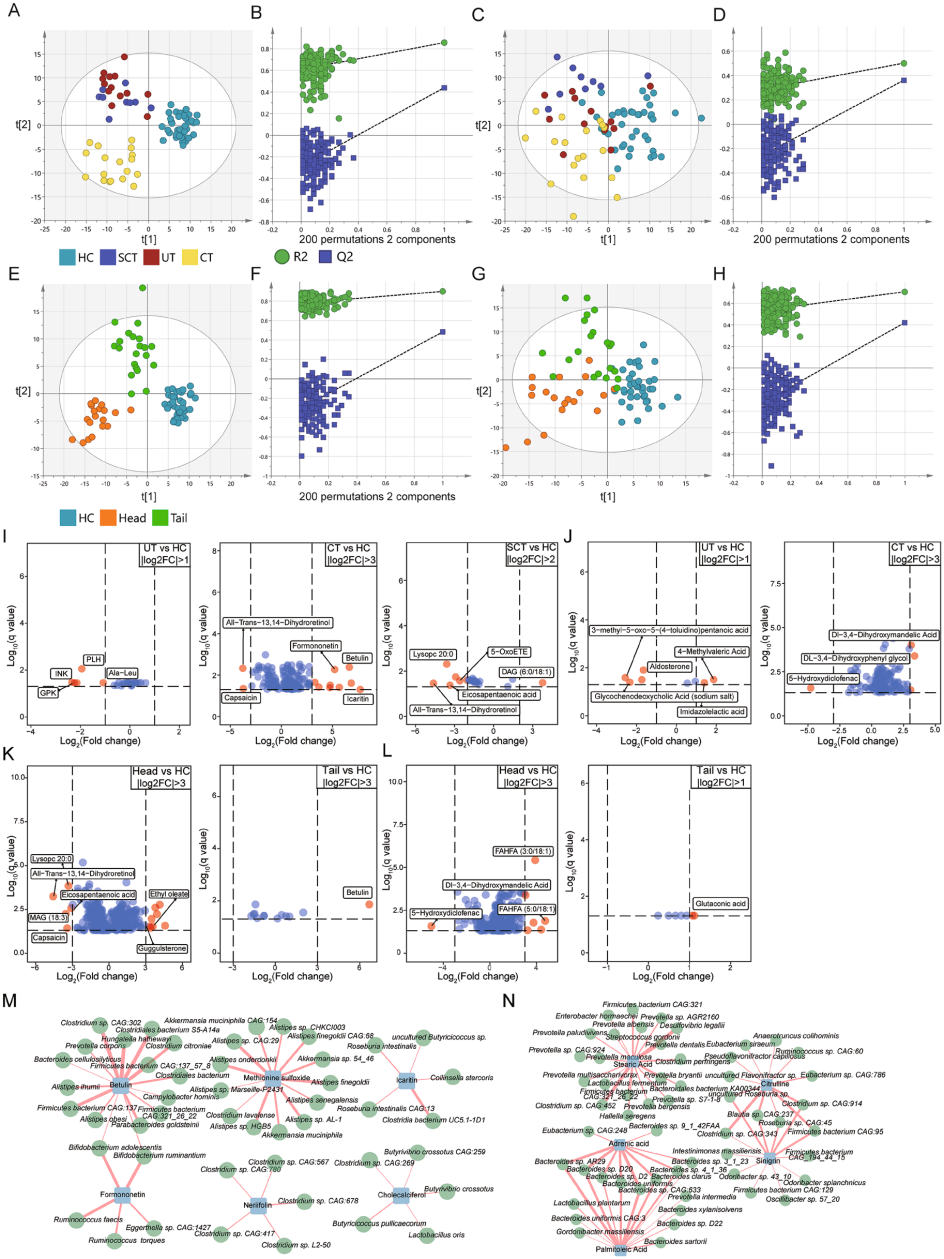
Untargeted metabolome analysis. **(A,C)** The orthogonal partial least squares discriminant analysis (OPLS-DA) scores plot for HC, UT, CT and SCT groups based on metabolites of positive and negative ion mode, respectively. **(B,D)** Permutation test (*n* = 200) for OPLS-DA model of panels **(A,C)**, respectively. **(E,G)** OPLS-DA scores plot for HC, Head and Tail groups based on metabolites of positive and negative ion mode, respectively. **(F,H)** Permutation test (*n* = 200) for OPLS-DA model of panels **(E,G)**, respectively. **(I)** The top-ranked differential metabolites of positive ion mode for UT, CT and SCT compared with HC. **(J)** The top-ranked differential metabolites of negative ion mode for UT and CT compared with HC. **(K,L)** The top-ranked differential metabolites of positive and negative ion mode for Head and Tail compared with HC, respectively. Differential metabolites with higher fold changes in comparison between groups are displayed with red dots (q < 0.05). **(M,N)** Correlation analysis between 810 abundant microbes and differential metabolites of interest in positive and negative ion mode, respectively (|r| > 0.4, q < 0.05).

To further investigate the alterations in fecal metabolites, untargeted metabolomic analysis was conducted on 79 fecal samples. Orthogonal partial least squares discriminant analysis (OPLS-DA) was conducted to examine the differences in metabolome across groups. In positive ion mode, three distinct clusters were identified among the four groups (Figure 5A). To evaluate potential overfitting, a permutation test was performed, generating 200 OPLS models by randomly permuting the categorical variables (Figure 5B). The permuted Q2 and R2 values consistently fell below the original values, indicating that the model is robust and reliable without overfitting (Wen et al., 2019). In negative ion mode, a clear separation between the CT and HC groups was also observed (Figure 5C). The slope of R2 was 0.285 (<0.4) and the intercept of Q2 was −0.258 (<0.05) (Figure 5D), confirming the absence of overfitting (Shi et al., 2020). Additionally, discriminant clusters were identified among the Head, Tail and HC groups based on positive and negative ion mode metabolites, separately (Figures 5E–H).

Differential analysis was conducted to identify discriminant metabolites in positive ion mode between the HC and other groups (VIP > 1, q < 0.05). The top-ranked differential metabolites in positive ion mode were displayed (Figure 5I). Notably, icaritin, known for its antitumor activities (Shi et al., 2020), was significantly up-regulated in CT group compared to HC. Formononetin and betulin, both of which exhibit antitumorigenic properties *in vitro* (Rzeski et al., 2009; Tay et al., 2019), were also significantly up-regulated in CT group. Methionine sulfoxide, the major product of methionine oxidation which activates pyruvate kinase M2 to promote pancreatic cancer metastasis (He et al., 2022), was upregulated in CT group. Indole-3-acetic acid, a modulator that enhances chemotherapy efficacy in pancreatic cancer (Tintelnot et al., 2023), was increased in CT group. Conversely, capsaicin, which has been shown to suppress the growth of pancreatic cancer (Zhang et al., 2008), was significantly decreased in CT group. Esculetin, which induces antiproliferative and apoptotic response in pancreatic cancer cells (Arora et al., 2016), was increased in CT group. S-Adenosylmethionine, which sensitizes pancreatic tumor cells to chemotherapeutic agents (Kesh et al., 2022), was decreased in UT group.

In Head group, 147 out of 331 differential metabolites detected in positive ion mode were elevated compared to HC group, whereas only 6 out of 13 differential metabolites showed increased levels in Tail group. The top-ranked differential metabolites in positive ion mode were displayed (Figure 5K). These results indicate that the Head group exhibits greater alterations in fecal metabolites relative to the HC group compared to Tail group.

To further investigate the correlations between differential metabolites and microbes, Pearson correlation analysis was conducted (Figure 5M). Results showed a positive correlation between icaritin and butyrate-producing bacteria, such as *Roseburia intestinalis*, *Roseburia intestinalis CAG:13*, and uncultured *Butyricicoccus* sp. (Kasahara et al., 2018). Previous studies have shown that butyrate enhances anti-tumor activity in syngeneic murine pancreatic cancer model (Luu et al., 2021). Additionally, probiotics associated with antitumor effects, such as *Bifidobacterium* spp. (Luu et al., 2021), were found to be positively correlated with betulin and formononetin. Short-chain fatty acid-producing probiotics, such as *Butyrivibrio crossotus*, *Butyricicoccus pullicaecorum* and *Lactobacillus oris*, showed positive correlations with cholecalciferol (vitamin D3). *Clostridium*, which has been identified as a promising agent for anticancer treatment (Yaghoubi et al., 2021), was positively correlated with neriifolin.

Differential analysis was conducted in negative ion mode to identify discriminant metabolites between the HC and other groups (VIP > 1, q < 0.05). The top-ranked differential metabolites in negative ion mode were displayed (Figure 5J). Notably, sinigrin, which has demonstrated anti-cancer, antibacterial and anti-inflammatory properties (Mazumder et al., 2016), was significantly upregulated in CT group. Glycerol-3-phosphate, which has been associated with improved survival rates in pancreatic cancer patients (Ohara et al., 2024), showed a significant increase in CT group. Mevalonic acid, an essential compound for the growth of pancreatic cancer cells (Sumi et al., 1992), was also significantly increased in the CT group. Gluconic acid, which holds remarkable potential for monitoring the progression and metastasis of pancreatic cancer (Luo et al., 2020), was significantly elevated in CT group.

In Head group, 146 out of 184 differential metabolites in negative ion mode were significantly elevated compared to HC, whereas only 7 differential metabolites were identified in Tail group. The top-ranked differential metabolites in negative ion mode were displayed (Figure 5L). Correlation analysis between microbes and metabolites in negative ion mode revealed that stearic acid was positively correlated with *Prevotella* spp., while *Bacteroides* spp. showed positive correlations with adrenic acid and palmitoleic acid (Figure 5N). Adrenic acid, identified as a potential biomarker for PDAC detection, exhibits excellent diagnostic performance (Cao et al., 2021). *Prevotella intermedia*, which has been associated with pancreatic cancer (Petrick et al., 2022), was positively correlated with palmitoleic acid. Study also has reported that overexpression of ZNF488 promotes pancreatic cancer cell proliferation and tumorigenesis by enhancing palmitoleic acid production (Xiao et al., 2023). *Odoribacter splanchnicus*, implicated in colorectal carcinogenesis (Png et al., 2022), was positively correlated with sinigrin.

### Identification of pancreatic cancer patients

**Figure 6:**
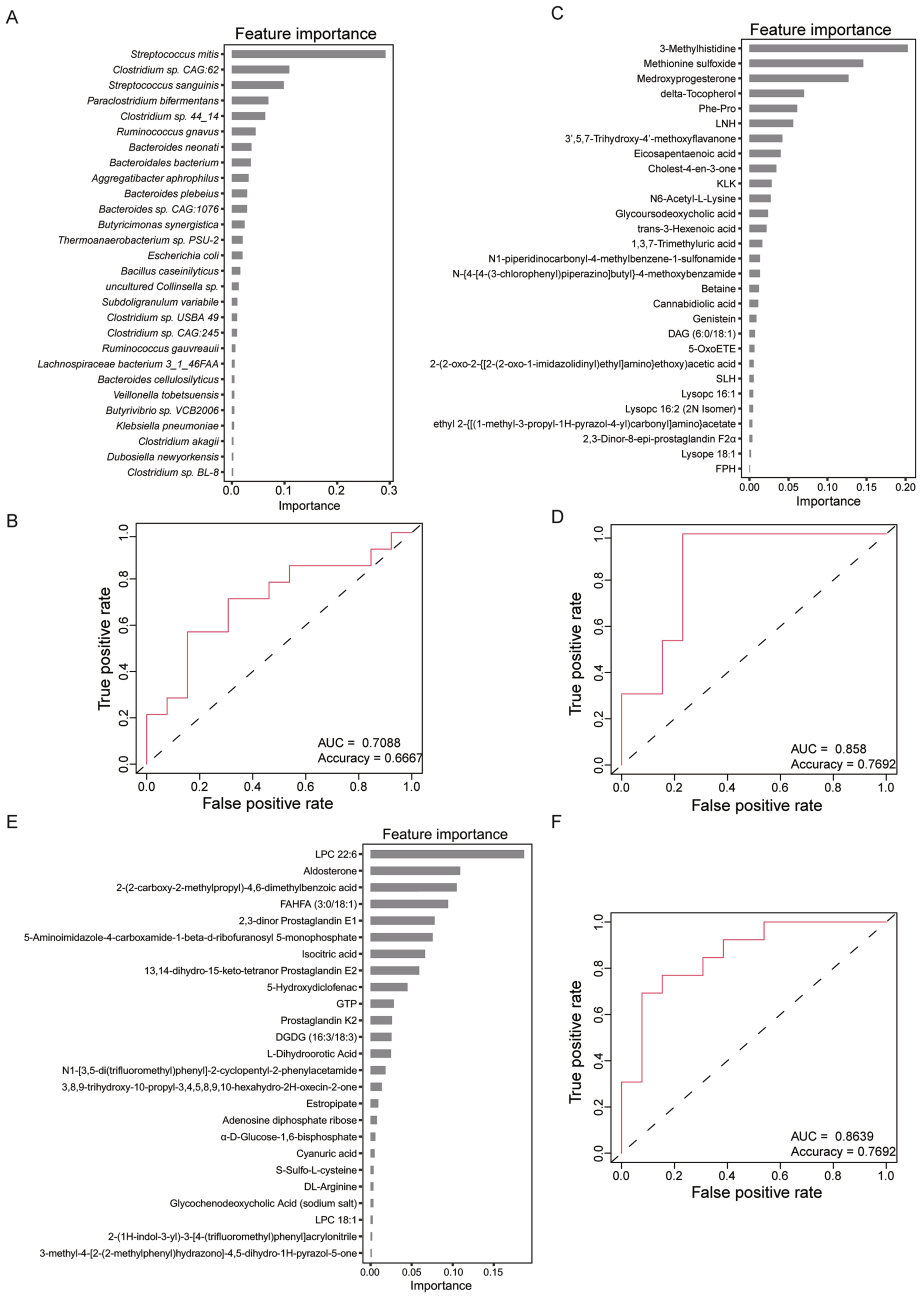
Classifiers for HC and pancreatic cancer patients using eXtreme Gradient Boosting (XGBoost) method. **(A)** The 28 optimal features of gut microbes selected for constructing classifier model. **(B)** Receiver operating characteristic (ROC) curve of the classifier based on 28 microbes. **(C)** The 29 optimal features of metabolites of the positive ion mode selected for constructing classifier model. **(D)** ROC curve of the classifier based on 29 metabolites of the positive ion mode. **(E)** The 25 optimal features of metabolites of the negative ion mode selected for constructing classifier model. **(F)** ROC curve of the classifier based on 25 metabolites of the negative ion mode.

We further developed a classifier for distinguish between HC and pancreatic cancer patients using the eXtreme Gradient Boosting (XGBoost) method. Classifier was constructed based on the composition of gut microbes and fecal metabolites. Specifically, 28 species were selected as the optimal features for model construction. Notably, *Streptococcus mitis*, which was significantly enriched in CT group, emerged as the most important feature (Figure 6A). By integrating the abundance data of 28 species, the model achieved an AUC of 0.71 and an accuracy of 0.67 in the validation dataset (Figure 6B).

Furthermore, another model was constructed using 29 metabolites identified in positive ion mode. Notably, methionine sulfoxide, which was significantly enriched in UT, CT and SCT groups, emerged as the second most important feature (Figure 6C). Based on 29 metabolites, the model achieved an AUC of 0.858 and an accuracy of 0.7692 in the validation dataset (Figure 6D). Additionally, a comparable model performance was obtained using 25 metabolites identified in the negative ion mode (Figure 6E), with similar discriminatory power to the positive ion mode model (Figure 6F). The superior discriminatory ability of fecal metabolites compared to microbial markers underscores their potential utility in identifying pancreatic cancer patients.

## Discussion

Our study revealed significant alterations in gut microbial composition between HC and pancreatic cancer patients with or without treatments. Notably, after excluding nPDAC patients, significant differences in gut microbiota were observed between the HC, UT, CT and SCT groups. However, no significant differences were found among the UT, CT and SCT groups. This suggests that the impact of pancreatic cancer itself on gut microbiota may be more substantial than the effect of treatment modalities. Additionally, most patients are ineligible for curative surgery, with chemotherapy and/or radiotherapy being the main treatment options, which often have limited efficacy (Merali et al., 2024). Consequently, the unchanged disease status post-chemotherapy may determine microbial composition.

At present, gut microbiota and their metabolites have garnered increasing attention in pancreatic cancer therapy. Several species associated with enhanced tumor immune response were observed in long-term survivors of pancreatic cancer (Kharofa et al., 2023). Studies indicated that microbiota-derived metabolite has clinical implications in the treatment of PDAC (Mirji et al., 2022; Tintelnot et al., 2023). Murine experiments indicated that alterations in gut microbiota could influence tumor microbiota and tumor growth (Cheng et al., 2025). Therefore, microbial signatures may serve as novel biomarkers for the treatment and prognosis of pancreatic cancer. In our study, we found that *Lactobacillus*, previously identified as beneficial for pancreatic cancer alleviation (Zhu et al., 2023), was enriched in CT and SCT groups. Specifically, *Lactobacillus rhamnosus* also has been shown to alleviate clinical symptoms and/or prevent intestinal disorders through both clinical studies and animal experiments (Huang et al., 2022). Therefore, it is worth investigating whether the therapeutic effects of CT and SCT on pancreatic cancer are partly mediated by increased abundance of *Lactobacillus*.

We found that *Streptococcu*, such as *Streptococcus mutans* and *Streptococcus pneumoniae*, were positively associated with both CT and Head groups. Notably, oral carriage of *Streptococcu* was associated with a higher risk of PDAC (Wei et al., 2020). Additionally, a two-sample Mendelian Randomization analysis identified *Streptococcu* as potential causative factors for PDAC (Jiang et al., 2023). The increased abundance of *Streptococcu* in CT group may be associated with the progression of pancreatic cancer. Furthermore, only six differentially abundant species between HC and Tail groups were identified, suggesting that gut microbial composition of the Tail group is more similar to that of the HC group. Importantly, *Veillonella* and opportunistic pathogens such as *Escherichia coli*, *Shigella sonnei*, *Shigella flexneri* and *Shigella dysenteriae* were positively associated with the Head group but negatively associated with the Tail group. Pancreatic cancer patients are particularly predisposed to developing *Escherichia coli* (*E. coli*) bloodstream infection (Bai et al., 2022). Moreover, multiple studies have demonstrated that four genera, such as *Escherichia*, *Shigella* and *Veillonella*, are significantly elevated in PDAC patients compared to HC (Hong et al., 2024). Therefore, we speculate that *Escherichia coli* infection in pancreatic cancer patients may be associated with the retrograde dissemination of gut increased *Escherichia coli*. Apart from the tumor location, other characteristics of the tumor are also associated with the gut microbiota, such as tumor size, and tumor stage (Yachida et al., 2019; Chen et al., 2022; Yang et al., 2023). In this study, we only focused on the associations between tumor location and gut microbiota. The relationship between other clinical variables and gut microbiota requires more research to reveal.

The elevated fecal UA levels in the Head group were associated with the increased abundance of *Shigella* spp. and *Escherichia coli*. Our previous research has established the associations of *Shigella* spp. and *Escherichia coli* with UA levels, supported by animal experiments that demonstrated the role of *Escherichia coli* in regulating host UA levels (Han et al., 2024). Consequently, the higher abundance of *Shigella* spp. and *Escherichia coli* likely contribute to the elevated UA levels in the Head group. Previous study has also reported an association between high serum UA levels and an increased risk of pancreatic cancer (Huang et al., 2020). Therefore, targeting *Shigella* spp. and *Escherichia coli* may represent a promising strategy for regulating host plasma UA levels and potentially preventing pancreatic cancer in future.

Several studies have indicated that pancreatic head cancer and pancreatic body/tail cancer are different diseases (Birnbaum et al., 2019; Sun et al., 2022). In our study, we found significant differences in microbial composition and SNP densities between the Head and Tail groups. What’s more, through analyzing 86 species, we found that SNPs of 27 species were associated with the Head group but not the Tail group. The SNP density of *Lachnospiraceae bacterium 2_1_58FAA* showed the highest significance and negatively associated with the Head group. Study has shown that the abundance of *Lachnospiraceae bacterium 2_1_58FAA* is positively associated with colorectal neoplasms (Wu et al., 2020). To our knowledge, our study is the first to reveal an association between SNPs of *Lachnospiraceae bacterium 2_1_58FAA* and pancreatic head cancer. Microbial SNPs account for considerable genetic variation, and some are linked to certain diseases. Previous study indicated that the 171S/L HtrA mutation in *Helicobacter pylori* promotes gastric cancer development and as a potential biomarker for risk predictions (Sharafutdinov et al., 2023). Also, bacterial SNPs in the human gut microbiome associate with host body mass index (Zahavi et al., 2023). However, whether individual SNPs in gut microbiota play an important role in pancreatic cancer development is unknown. Our results provide supports for further elucidation of the causal link between microbial SNPs and pancreatic cancer. On this basis, targeting individual strains or mutation sites may be an effective strategy to personalized treatment and improve efficacy.

Totally, we assembled 723 nrMAGs using all samples, and obtained 276 high-quality and 424 medium-quality nrMAGs. Among these, 26 SGBs were classified as unknown SGBs. Given the limitations of read-based taxonomic analysis due to incomplete reference databases, numerous studies have constructed microbial genome catalogs, such as those related to COVID-19 (Ke et al., 2022) and Hadza hunter-gatherers (Carter et al., 2023). To our knowledge, this is the first microbial genome catalog specifically for pancreatic cancer patients. We identified thioamitides produced by MAG100 (Methanobrevibacter) and lassopeptide produced by MAG277 (Clostridium) and MAG331 (Bacteroidales), which show great potential in anticancer drug development (Cheng and Hua, 2020; Eyles et al., 2021). However, the three MAGs could not be annotated to the species level based on existing databases, hindering mechanistic studies. *De novo* assembly and binning to reconstruct MAGs for pancreatic cancer patients enable the study of organisms absent from reference databases. Our results provide a valuable reference dataset for further study of gut microbiota in patients with pancreatic cancer.

We identified several fecal metabolites with antitumor activity that were significantly elevated in CT group, including icaritin, formononetin, betulin and sinigrin. Notably, icaritin exhibited a positive correlation with butyrate-producing bacteria, whereas a decrease in butyrate-producing bacteria in pancreatic cancer patients was revealed (Sono et al., 2024). The elevated levels of icaritin in CT group may be associated with chemotherapy. Conversely, methionine oxidation metabolites, which promote pancreatic cancer metastasis (He et al., 2022), and mevalonic acid, essential for the growth of pancreatic cancer cells (Sumi et al., 1992), were increased in CT group. Although chemotherapy increases certain anti-tumor related fecal metabolites, metabolites that promote tumor metastasis and growth are still increased, potentially contributing to the limited efficacy of chemotherapy (Merali et al., 2024). Additionally, short-chain fatty acid-producing probiotics, such as *Butyrivibrio crossotus*, *Butyricicoccus pullicaecorum* and *Lactobacillus oris*, were positively correlated with cholecalciferol (vitamin D3). Numerous epidemiological and clinical studies suggest that higher vitamin D intake is associated with a lower risk of pancreatic cancer (Skinner et al., 2006; Altieri et al., 2017). The relationship between short-chain fatty acid-producing probiotics and vitamin D in pancreatic cancer patients warrants further investigation.

The early diagnosis of pancreatic cancer remains a challenge. Some studies have found that PDAC biomarkers from urine (Blyuss et al., 2020) and serum (Seifert et al., 2020) have limitations in the diagnosis of pancreatic cancer. Previous study results indicated that fecal microbiota screening for the early detection of PDAC is feasible (Kartal et al., 2022). In our study, the diagnostic model based on fecal metabolites outperformed the one based on gut microbiota, highlighting the potential of fecal metabolites in pancreatic cancer diagnosis. Also, the differences in gut microbial composition between HC and pancreatic cancer patients were minimal with only weak statistical significance. However, the fecal metabolome exhibited more pronounced differences between HC and pancreatic cancer patients. Previous research has demonstrated that a risk prediction model utilizing circulating microbial and non-microbial metabolites can serve as a promising tool for identifying individuals at high risk of pancreatic cancer (Irajizad et al., 2023). Our study further underscores the diagnostic potential of fecal metabolites in pancreatic cancer. The development of a screening or diagnostic tool for pancreatic cancer based on fecal metabolites is worthy of further testing.

## Conclusion

Our study offers an in-depth and comprehensive analysis of the gut microbiota in pancreatic cancer patients, encompassing both treatment-naïve individuals and those who have undergone chemotherapy and surgery combined with chemotherapy. Notably, our research is the first to uncover associations between microbial SNPs and pancreatic head cancer. We construct the first gut microbial genome catalog specific to pancreatic cancer patients, and revealed that fecal metabolites hold potential for pancreatic cancer diagnosis. This study enhances our understanding of the relationship between gut microbiota and pancreatic cancer and paves the way for the development of microbiota-based precision therapies for this malignancy.

## Data Availability

The raw metagenomic sequencing dataset reported in this study has been deposited in the Genome Sequence Archive in National Genomics Data Center (GSA: CRA021840) that are publicly accessible at https://ngdc.cncb.ac.cn/gsa. The metadata of all samples included in this study is presented in Supplementary Table 1.

## Footnotes

^1^ https://github.com/BGI-shenzhen/VCF2Dis

## References

[ref1] Alneberg J. Bjarnason B. S. de Bruijn I. Schirmer M. Quick J. Ijaz U. Z.. (2014). Binning metagenomic contigs by coverage and composition. Nat. Methods 11, 1144–1146. doi: 10.1038/nmeth.3103, PMID: 25218180

[ref2] Altieri B. Grant W. B. Della Casa S. Orio F. Pontecorvi A. Colao A.. (2017). Vitamin D and pancreas: the role of sunshine vitamin in the pathogenesis of diabetes mellitus and pancreatic cancer. Crit. Rev. Food Sci. Nutr. 57, 3472–3488. doi: 10.1080/10408398.2015.1136922, PMID: 27030935

[ref3] Arora R. Sawney S. Saini V. Steffi C. Tiwari M. Saluja D. (2016). Esculetin induces antiproliferative and apoptotic response in pancreatic cancer cells by directly binding to KEAP1. Mol. Cancer 15:64. doi: 10.1186/s12943-016-0550-2, PMID: 27756327 PMC5069780

[ref4] Asnicar F. Thomas A. M. Beghini F. Mengoni C. Manara S. Manghi P.. (2020). Precise phylogenetic analysis of microbial isolates and genomes from metagenomes using PhyloPhlAn 3.0. Nat. Commun. 11:2500. doi: 10.1038/s41467-020-16366-7, PMID: 32427907 PMC7237447

[ref5] Bai C. Zhang X. Yang D. Li D. Feng H. Li Y. (2022). Clinical analysis of bloodstream infection of *Escherichia coli* in patients with pancreatic Cancer from 2011 to 2019. Can. J. Infect. Dis. Med. Microbiol. 2022, 1–8. doi: 10.1155/2022/1338188, PMID: 35340919 PMC8942694

[ref6] Bethlehem L. van Echten-Deckert G. (2021). Ectoines as novel anti-inflammatory and tissue protective lead compounds with special focus on inflammatory bowel disease and lung inflammation. Pharmacol. Res. 164:105389. doi: 10.1016/j.phrs.2020.105389 33352226

[ref7] Birnbaum D. J. Bertucci F. Finetti P. Birnbaum D. Mamessier E. (2019). Head and body/tail pancreatic carcinomas are not the same tumors. Cancers 11:497. doi: 10.3390/cancers11040497, PMID: 30965637 PMC6520848

[ref8] Blin K. Shaw S. Augustijn H. E. Reitz Z. L. Biermann F. Alanjary M.. (2023). antiSMASH 7.0: new and improved predictions for detection, regulation, chemical structures and visualisation. Nucleic Acids Res. 51, W46–w50. doi: 10.1093/nar/gkad344, PMID: 37140036 PMC10320115

[ref9] Blyuss O. Zaikin A. Cherepanova V. Munblit D. Kiseleva E. M. Prytomanova O. M.. (2020). Development of PancRISK, a urine biomarker-based risk score for stratified screening of pancreatic cancer patients. Br. J. Cancer 122, 692–696. doi: 10.1038/s41416-019-0694-0, PMID: 31857725 PMC7054390

[ref10] Cao Y. Zhao R. Guo K. Ren S. Zhang Y. Lu Z.. (2021). Potential metabolite biomarkers for early detection of stage-I pancreatic ductal adenocarcinoma. Front. Oncol. 11:744667. doi: 10.3389/fonc.2021.744667, PMID: 35127469 PMC8807510

[ref11] Carter M. M. Olm M. R. Merrill B. D. Dahan D. Tripathi S. Spencer S. P.. (2023). Ultra-deep sequencing of Hadza hunter-gatherers recovers vanishing gut microbes. Cell 186, 3111–3124.e13. doi: 10.1016/j.cell.2023.05.046, PMID: 37348505 PMC10330870

[ref12] Chen C. Du Y. Liu Y. Shi Y. Niu Y. Jin G.. (2022). Characteristics of gastric cancer gut microbiome according to tumor stage and age segmentation. Appl. Microbiol. Biotechnol. 106, 6671–6687. doi: 10.1007/s00253-022-12156-x, PMID: 36083304

[ref13] Chen T. Guestrin C. (2016) XGBoost: a scalable tree boosting system. In *Proceedings of the 22nd ACM SIGKDD international conference on knowledge discovery and data mining*. San Francisco, California, USA: Association for Computing Machinery, pp. 785–794.

[ref14] Chen Y. Liu P. Liu R. Hu S. He Z. Dong G.. (2021). Comprehensive strain-level analysis of the gut microbe *Faecalibacterium prausnitzii* in patients with liver cirrhosis. mSystems 6:e0077521. doi: 10.1128/msystems.00775-21, PMID: 34342541 PMC8407477

[ref15] Cheng H. Guo H. Wen C. Sun G. Tang F. Li Y. (2025). The dual role of gut microbiota in pancreatic cancer: new insights into onset and treatment. Ther Adv Med Oncol 17:17588359251324882. doi: 10.1177/17588359251324882, PMID: 40093983 PMC11909682

[ref16] Cheng C. Hua Z. C. (2020). Lasso peptides: heterologous production and potential medical application. Front. Bioeng. Biotechnol. 8:571165. doi: 10.3389/fbioe.2020.571165, PMID: 33117783 PMC7549694

[ref17] Cingolani P. Platts A. Wang le L. Coon M. Nguyen T. Wang L.. (2012). A program for annotating and predicting the effects of single nucleotide polymorphisms, SnpEff: SNPs in the genome of *Drosophila melanogaster* strain w1118; iso-2; iso-3. Fly 6, 80–92. doi: 10.4161/fly.19695, PMID: 22728672 PMC3679285

[ref18] Eyles T. H. Vior N. M. Lacret R. Truman A. W. (2021). Understanding thioamitide biosynthesis using pathway engineering and untargeted metabolomics. Chem. Sci. 12, 7138–7150. doi: 10.1039/D0SC06835G, PMID: 34123341 PMC8153245

[ref19] Fang Q. Lai Y. Zhang D. Lei H. Wang F. Guo X.. (2023). Gut microbiota regulation and prebiotic properties of polysaccharides from Oudemansiella raphanipes mushroom. World J. Microbiol. Biotechnol. 39:167. doi: 10.1007/s11274-023-03616-1, PMID: 37076579

[ref20] Han Y. Liu X. Jia Q. Xu J. Shi J. Li X.. (2024). Longitudinal multi-omics analysis uncovers the altered landscape of gut microbiota and plasma metabolome in response to high altitude. Microbiome 12:70. doi: 10.1186/s40168-024-01781-5, PMID: 38581016 PMC10996103

[ref21] He D. Feng H. Sundberg B. Yang J. Powers J. Christian A. H.. (2022). Methionine oxidation activates pyruvate kinase M2 to promote pancreatic cancer metastasis. Mol. Cell 82, 3045–3060.e11. doi: 10.1016/j.molcel.2022.06.005, PMID: 35752173 PMC9391305

[ref22] Hong J. Fu Y. Chen X. Zhang Y. Li X. Li T.. (2024). Gut microbiome changes associated with chronic pancreatitis and pancreatic cancer: a systematic review and meta-analysis. Int. J. Surg. 110, 5781–5794. doi: 10.1097/JS9.0000000000001724, PMID: 38847785 PMC11392207

[ref23] Hu L. Qiao Y. Liu J. Zheng C. Wang X. Sun P.. (2022). Characterization of histidine functionalization and its timing in the biosynthesis of Ribosomally synthesized and Posttranslationally modified Thioamitides. J. Am. Chem. Soc. 144, 4431–4438. doi: 10.1021/jacs.1c11669, PMID: 35230829

[ref24] Huang C. F. Huang J. J. Mi N. N. Lin Y. Y. He Q. S. Lu Y. W.. (2020). Associations between serum uric acid and hepatobiliary-pancreatic cancer: a cohort study. World J. Gastroenterol. 26, 7061–7075. doi: 10.3748/wjg.v26.i44.7061, PMID: 33311950 PMC7701939

[ref25] Huang R. Wu F. Zhou Q. Wei W. Yue J. Xiao B.. (2022). Lactobacillus and intestinal diseases: mechanisms of action and clinical applications. Microbiol. Res. 260:127019. doi: 10.1016/j.micres.2022.127019, PMID: 35421680

[ref26] Irajizad E. Kenney A. Tang T. Vykoukal J. Wu R. Murage E.. (2023). A blood-based metabolomic signature predictive of risk for pancreatic cancer. Cell Rep Med 4:101194. doi: 10.1016/j.xcrm.2023.101194, PMID: 37729870 PMC10518621

[ref27] Jiang Z. Mou Y. Wang H. Li L. Jin T. Wang H.. (2023). Causal effect between gut microbiota and pancreatic cancer: a two-sample Mendelian randomization study. BMC Cancer 23:1091. doi: 10.1186/s12885-023-11493-y, PMID: 37950180 PMC10636952

[ref28] Kaiki Y. Kitagawa H. Tadera K. Taogoshi H. Ikeda M. Kano M.. (2021). Laboratory identification and clinical characteristics of *Streptococcus bovis*/*Streptococcus equinus* complex bacteremia: a retrospective, multicenter study in Hiroshima Japan. BMC Infect. Dis. 21:1192. doi: 10.1186/s12879-021-06880-4, PMID: 34836500 PMC8626886

[ref29] Kang D. D. Froula J. Egan R. Wang Z. (2015). MetaBAT, an efficient tool for accurately reconstructing single genomes from complex microbial communities. PeerJ 3:e1165. doi: 10.7717/peerj.1165, PMID: 26336640 PMC4556158

[ref30] Kartal E. Schmidt T. S. B. Molina-Montes E. Rodríguez-Perales S. Wirbel J. Maistrenko O. M.. (2022). A faecal microbiota signature with high specificity for pancreatic cancer. Gut 71, 1359–1372. doi: 10.1136/gutjnl-2021-324755, PMID: 35260444 PMC9185815

[ref31] Kasahara K. Krautkramer K. A. Org E. Romano K. A. Kerby R. L. Vivas E. I.. (2018). Interactions between Roseburia intestinalis and diet modulate atherogenesis in a murine model. Nat. Microbiol. 3, 1461–1471. doi: 10.1038/s41564-018-0272-x, PMID: 30397344 PMC6280189

[ref32] Ke S. Weiss S. T. Liu Y. Y. (2022). Dissecting the role of the human microbiome in COVID-19 via metagenome-assembled genomes. Nat. Commun. 13:5235. doi: 10.1038/s41467-022-32991-w, PMID: 36068270 PMC9446638

[ref33] Kesh K. Mendez R. Mateo-Victoriano B. Garrido V. T. Durden B. Gupta V. K.. (2022). Obesity enriches for tumor protective microbial metabolites and treatment refractory cells to confer therapy resistance in PDAC. Gut Microbes 14:2096328. doi: 10.1080/19490976.2022.2096328, PMID: 35816618 PMC9275504

[ref34] Kharofa J. Haslam D. Wilkinson R. Weiss A. Patel S. Wang K.. (2023). Analysis of the fecal metagenome in long-term survivors of pancreas cancer. Cancer 129, 1986–1994. doi: 10.1002/cncr.34748, PMID: 36943918

[ref35] Lefort V. Desper R. Gascuel O. (2015). FastME 2.0: a comprehensive, accurate, and fast distance-based phylogeny inference program. Mol. Biol. Evol. 32, 2798–2800. doi: 10.1093/molbev/msv150, PMID: 26130081 PMC4576710

[ref36] León-Letelier R. A. Dou R. Vykoukal J. Yip-Schneider M. T. Maitra A. Irajizad E.. (2024). Contributions of the microbiome-derived metabolome for risk assessment and prognostication of pancreatic Cancer. Clin. Chem. 70, 102–115. doi: 10.1093/clinchem/hvad186, PMID: 38175578 PMC11836914

[ref37] Letunic I. Bork P. (2024). Interactive tree of life (iTOL) v6: recent updates to the phylogenetic tree display and annotation tool. Nucleic Acids Res. 52, W78–w82. doi: 10.1093/nar/gkae268, PMID: 38613393 PMC11223838

[ref38] Liang Y. Du M. Li X. Gao J. Li Q. Li H.. (2025). Upregulation of Lactobacillus spp. in gut microbiota as a novel mechanism for environmental eustress-induced anti-pancreatic cancer effects. Gut Microbes 17:2470372. doi: 10.1080/19490976.2025.2470372, PMID: 39988618 PMC11853549

[ref39] Luo X. Liu J. Wang H. Lu H. (2020). Metabolomics identified new biomarkers for the precise diagnosis of pancreatic cancer and associated tissue metastasis. Pharmacol. Res. 156:104805. doi: 10.1016/j.phrs.2020.104805 32278036

[ref40] Luu M. Riester Z. Baldrich A. Reichardt N. Yuille S. Busetti A.. (2021). Microbial short-chain fatty acids modulate CD8(+) T cell responses and improve adoptive immunotherapy for cancer. Nat. Commun. 12:4077. doi: 10.1038/s41467-021-24331-1, PMID: 34210970 PMC8249424

[ref41] Ma C. Chen K. Wang Y. Cen C. Zhai Q. Zhang J. (2021a). Establishing a novel colorectal cancer predictive model based on unique gut microbial single nucleotide variant markers. Gut Microbes 13, 1–6. doi: 10.1080/19490976.2020.1869505, PMID: 33430705 PMC7808391

[ref42] Ma C. Zhang C. Chen D. Jiang S. Shen S. Huo D.. (2021b). Probiotic consumption influences universal adaptive mutations in indigenous human and mouse gut microbiota. Commun Biol 4:1198. doi: 10.1038/s42003-021-02724-8, PMID: 34663913 PMC8523657

[ref43] Mallick H. Rahnavard A. McIver L. J. Ma S. Zhang Y. Nguyen L. H.. (2021). Multivariable association discovery in population-scale meta-omics studies. PLoS Comput. Biol. 17:e1009442. doi: 10.1371/journal.pcbi.1009442, PMID: 34784344 PMC8714082

[ref44] Mazumder A. Dwivedi A. du Plessis J. (2016). Sinigrin and its therapeutic benefits. Molecules 21:416. doi: 10.3390/molecules21040416, PMID: 27043505 PMC6273501

[ref45] McGuigan A. Kelly P. Turkington R. C. Jones C. Coleman H. G. McCain R. S. (2018). Pancreatic cancer: a review of clinical diagnosis, epidemiology, treatment and outcomes. World J. Gastroenterol. 24, 4846–4861. doi: 10.3748/wjg.v24.i43.4846, PMID: 30487695 PMC6250924

[ref46] Mei Q. X. Huang C. L. Luo S. Z. Zhang X. M. Zeng Y. Lu Y. Y. (2018). Characterization of the duodenal bacterial microbiota in patients with pancreatic head cancer vs. healthy controls. Pancreatology 18, 438–445. doi: 10.1016/j.pan.2018.03.005, PMID: 29653723

[ref47] Merali N. Chouari T. Sweeney C. Halle-Smith J. Maria-Danae J. Wang B.. (2024). The microbial composition of pancreatic ductal adenocarcinoma: a systematic review of 16S rRNA gene sequencing. Int. J. Surg. 110, 6771–6799. doi: 10.1097/JS9.0000000000001762, PMID: 38874485 PMC11487005

[ref48] Mirji G. Worth A. Bhat S. A. El Sayed M. Kannan T. Goldman A. R.. (2022). The microbiome-derived metabolite TMAO drives immune activation and boosts responses to immune checkpoint blockade in pancreatic cancer. Sci Immunol 7:eabn0704. doi: 10.1126/sciimmunol.abn0704, PMID: 36083892 PMC9925043

[ref49] Nagata N. Nishijima S. Kojima Y. Hisada Y. Imbe K. Miyoshi-Akiyama T.. (2022). Metagenomic identification of microbial signatures predicting pancreatic Cancer from a multinational study. Gastroenterology 163, 222–238. doi: 10.1053/j.gastro.2022.03.054, PMID: 35398347

[ref50] Nomura R. Matayoshi S. Otsugu M. Kitamura T. Teramoto N. Nakano K. (2020). Contribution of severe dental caries induced by *Streptococcus mutans* to the pathogenicity of infective endocarditis. Infect. Immun. 88:19. doi: 10.1128/IAI.00897-19, PMID: 32312765 PMC7309618

[ref51] Ohara Y. Craig A. J. Liu H. Yang S. Moreno P. Dorsey T. H.. (2024). LMO3 is a suppressor of the basal-like/squamous subtype and reduces disease aggressiveness of pancreatic cancer through glycerol 3-phosphate metabolism. Carcinogenesis 45, 475–486. doi: 10.1093/carcin/bgae011, PMID: 38366633 PMC11229528

[ref52] Olm M. R. Brown C. T. Brooks B. Banfield J. F. (2017). dRep: a tool for fast and accurate genomic comparisons that enables improved genome recovery from metagenomes through de-replication. ISME J. 11, 2864–2868. doi: 10.1038/ismej.2017.126, PMID: 28742071 PMC5702732

[ref53] Olm M. R. Crits-Christoph A. Bouma-Gregson K. Firek B. A. Morowitz M. J. Banfield J. F. (2021). inStrain profiles population microdiversity from metagenomic data and sensitively detects shared microbial strains. Nat. Biotechnol. 39, 727–736. doi: 10.1038/s41587-020-00797-0, PMID: 33462508 PMC9223867

[ref54] Parks D. H. Imelfort M. Skennerton C. T. Hugenholtz P. Tyson G. W. (2015). CheckM: assessing the quality of microbial genomes recovered from isolates, single cells, and metagenomes. Genome Res. 25, 1043–1055. doi: 10.1101/gr.186072.114, PMID: 25977477 PMC4484387

[ref55] Patro R. Duggal G. Love M. I. Irizarry R. A. Kingsford C. (2017). Salmon provides fast and bias-aware quantification of transcript expression. Nat. Methods 14, 417–419. doi: 10.1038/nmeth.4197, PMID: 28263959 PMC5600148

[ref56] Petrick J. L. Wilkinson J. E. Michaud D. S. Cai Q. Gerlovin H. Signorello L. B.. (2022). The oral microbiome in relation to pancreatic cancer risk in African Americans. Br. J. Cancer 126, 287–296. doi: 10.1038/s41416-021-01578-5, PMID: 34718358 PMC8770575

[ref57] Png C. W. Chua Y. K. Law J. H. Zhang Y. Tan K. K. (2022). Alterations in co-abundant bacteriome in colorectal cancer and its persistence after surgery: a pilot study. Sci. Rep. 12:9829. doi: 10.1038/s41598-022-14203-z, PMID: 35701595 PMC9198081

[ref58] Rzeski W. Stepulak A. Szymański M. Juszczak M. Grabarska A. Sifringer M.. (2009). Betulin elicits anti-cancer effects in tumour primary cultures and cell lines in vitro. Basic Clin. Pharmacol. Toxicol. 105, 425–432. doi: 10.1111/j.1742-7843.2009.00471.x, PMID: 19821831

[ref59] Saheb Kashaf S. Almeida A. Segre J. A. Finn R. D. (2021). Recovering prokaryotic genomes from host-associated, short-read shotgun metagenomic sequencing data. Nat. Protoc. 16, 2520–2541. doi: 10.1038/s41596-021-00508-2, PMID: 33864056

[ref60] Seifert A. M. Reiche C. Heiduk M. Tannert A. Meinecke A. C. Baier S.. (2020). Detection of pancreatic ductal adenocarcinoma with galectin-9 serum levels. Oncogene 39, 3102–3113. doi: 10.1038/s41388-020-1186-7, PMID: 32055023 PMC7142017

[ref61] Sharafutdinov I. Tegtmeyer N. Linz B. Rohde M. Vieth M. Tay A. C.. (2023). A single-nucleotide polymorphism in *Helicobacter pylori* promotes gastric cancer development. Cell Host Microbe 31, 1345–1358.e6. doi: 10.1016/j.chom.2023.06.016, PMID: 37490912

[ref62] Shi Z. J. Dimitrov B. Zhao C. Nayfach S. Pollard K. S. (2022). Fast and accurate metagenotyping of the human gut microbiome with GT-pro. Nat. Biotechnol. 40, 507–516. doi: 10.1038/s41587-021-01102-3, PMID: 34949778

[ref63] Shi R. Zhang J. Fang B. Tian X. Feng Y. Cheng Z.. (2020). Runners' metabolomic changes following marathon. Nutr. Metab. 17:19. doi: 10.1186/s12986-020-00436-0, PMID: 32190096 PMC7071712

[ref64] Sidiropoulos T. Dovrolis N. Katifelis H. Michalopoulos N. V. Kokoropoulos P. Arkadopoulos N.. (2024). Dysbiosis signature of fecal microbiota in patients with pancreatic adenocarcinoma and pancreatic Intraductal papillary mucinous neoplasms. Biomedicines 12:1040. doi: 10.3390/biomedicines12051040 38791002 PMC11117863

[ref65] Skinner H. G. Michaud D. S. Giovannucci E. Willett W. C. Colditz G. A. Fuchs C. S. (2006). Vitamin D intake and the risk for pancreatic cancer in two cohort studies. Cancer Epidemiol. Biomarkers Prev. 15, 1688–1695. doi: 10.1158/1055-9965.EPI-06-0206, PMID: 16985031

[ref66] Sono M. Iimori K. Nagao M. Ogawa S. Maruno T. Nakanishi Y.. (2024). Reduction of butyrate-producing bacteria in the gut microbiome of Japanese patients with pancreatic cancer. Pancreatology 24, 1031–1039. doi: 10.1016/j.pan.2024.09.002 39256134

[ref67] Sumi S. Beauchamp R. D. Townsend C. M. Jr. Uchida T. Murakami M. Rajaraman S.. (1992). Inhibition of pancreatic adenocarcinoma cell growth by lovastatin. Gastroenterology 103, 982–989. doi: 10.1016/0016-5085(92)90032-T, PMID: 1499946

[ref68] Sun K. Mylavarapu C. Crenshaw A. Zhang Y. Hsu E. Xu J.. (2022). Pancreatic head vs pancreatic body/tail cancer: are they different? World J Gastrointest Oncol 14, 716–723. doi: 10.4251/wjgo.v14.i3.716, PMID: 35321276 PMC8919010

[ref69] Tay K. C. Tan L. T. Chan C. K. Hong S. L. Chan K. G. Yap W. H.. (2019). Formononetin: a review of its anticancer potentials and mechanisms. Front. Pharmacol. 10:820. doi: 10.3389/fphar.2019.00820, PMID: 31402861 PMC6676344

[ref70] Tintelnot J. Xu Y. Lesker T. R. Schönlein M. Konczalla L. Giannou A. D.. (2023). Microbiota-derived 3-IAA influences chemotherapy efficacy in pancreatic cancer. Nature 615, 168–174. doi: 10.1038/s41586-023-05728-y, PMID: 36813961 PMC9977685

[ref71] Uritskiy G. V. DiRuggiero J. Taylor J. (2018). MetaWRAP—a flexible pipeline for genome-resolved metagenomic data analysis. Microbiome 6:158. doi: 10.1186/s40168-018-0541-1, PMID: 30219103 PMC6138922

[ref72] Wei A. L. Li M. Li G. Q. Wang X. Hu W. M. Li Z. L.. (2020). Oral microbiome and pancreatic cancer. World J. Gastroenterol. 26, 7679–7692. doi: 10.3748/wjg.v26.i48.7679, PMID: 33505144 PMC7789059

[ref73] Wei M. Y. Shi S. Liang C. Meng Q. C. Hua J. Zhang Y. Y.. (2019). The microbiota and microbiome in pancreatic cancer: more influential than expected. Mol. Cancer 18:97. doi: 10.1186/s12943-019-1008-0, PMID: 31109338 PMC6526613

[ref74] Weiser J. N. Ferreira D. M. Paton J. C. (2018). *Streptococcus pneumoniae*: transmission, colonization and invasion. Nat. Rev. Microbiol. 16, 355–367. doi: 10.1038/s41579-018-0001-8, PMID: 29599457 PMC5949087

[ref75] Wen X. Hu Y. Zhang X. Wei X. Wang T. Yin S. (2019). Integrated application of multi-omics provides insights into cold stress responses in pufferfish Takifugu fasciatus. BMC Genomics 20:563. doi: 10.1186/s12864-019-5915-7, PMID: 31286856 PMC6615287

[ref76] Wu Y. W. Simmons B. A. Singer S. W. (2016). MaxBin 2.0: an automated binning algorithm to recover genomes from multiple metagenomic datasets. Bioinformatics 32, 605–607. doi: 10.1093/bioinformatics/btv638, PMID: 26515820

[ref77] Wu S. Sun C. Li Y. Wang T. Jia L. Lai S.. (2020). GMrepo: a database of curated and consistently annotated human gut metagenomes. Nucleic Acids Res. 48, D545–d553. doi: 10.1093/nar/gkz764, PMID: 31504765 PMC6943048

[ref78] Xiao Q. Lan Z. Zhang S. Ren H. Wang S. Wang P.. (2023). Overexpression of ZNF488 supports pancreatic cancer cell proliferation and tumorigenesis through inhibition of ferroptosis via regulating SCD1-mediated unsaturated fatty acid metabolism. Biol. Direct 18:77. doi: 10.1186/s13062-023-00421-6, PMID: 37986084 PMC10658979

[ref79] Yachida S. Mizutani S. Shiroma H. Shiba S. Nakajima T. Sakamoto T.. (2019). Metagenomic and metabolomic analyses reveal distinct stage-specific phenotypes of the gut microbiota in colorectal cancer. Nat. Med. 25, 968–976. doi: 10.1038/s41591-019-0458-7, PMID: 31171880

[ref80] Yaghoubi A. Ghazvini K. Khazaei M. Hasanian S. M. Avan A. Soleimanpour S. J. R.. (2021). The use of Clostridium in cancer therapy: a promising way. Rev Res Med Microbiol. 33, 121–127. doi: 10.1097/MRM.0000000000000281

[ref81] Yang Q. Wang B. Zheng Q. Li H. Meng X. Zhou F.. (2023). A review of gut microbiota-derived metabolites in tumor progression and Cancer therapy. Adv Sci 10:e2207366. doi: 10.1002/advs.202207366, PMID: 36951547 PMC10214247

[ref82] Yu G. (2022). Data integration, manipulation and visualization of phylogenetic Trees. Chapman & Hall: Routledge.

[ref83] Yun W. G. Kim D. Lee M. Han Y. Chae Y. S. Jung H. S.. (2024). Comparing clinical and genomic features based on the tumor location in patients with resected pancreatic cancer. BMC Cancer 24:1048. doi: 10.1186/s12885-024-12795-5, PMID: 39187784 PMC11346014

[ref84] Zahavi L. Lavon A. Reicher L. Shoer S. Godneva A. Leviatan S.. (2023). Bacterial SNPs in the human gut microbiome associate with host BMI. Nat. Med. 29, 2785–2792. doi: 10.1038/s41591-023-02599-8, PMID: 37919437 PMC10999242

[ref85] Zhang R. Humphreys I. Sahu R. P. Shi Y. Srivastava S. K. (2008). In vitro and in vivo induction of apoptosis by capsaicin in pancreatic cancer cells is mediated through ROS generation and mitochondrial death pathway. Apoptosis 13, 1465–1478. doi: 10.1007/s10495-008-0278-6, PMID: 19002586

[ref86] Zhou W. Zhang D. Li Z. Jiang H. Li J. Ren R.. (2021). The fecal microbiota of patients with pancreatic ductal adenocarcinoma and autoimmune pancreatitis characterized by metagenomic sequencing. J. Transl. Med. 19:215. doi: 10.1186/s12967-021-02882-7, PMID: 34006295 PMC8130326

[ref87] Zhu Z. Yi B. Tang Z. Chen X. Li M. Xu T.. (2023). *Lactobacillus casei* combined with *Lactobacillus reuteri* alleviate pancreatic cancer by inhibiting TLR4 to promote macrophage M1 polarization and regulate gut microbial homeostasis. BMC Cancer 23:1044. doi: 10.1186/s12885-023-11557-z, PMID: 37904102 PMC10614400

